# Children and Adolescents’ Psychological Well-Being Became Worse in Heavily Hit Chinese Provinces during the COVID-19 Epidemic

**DOI:** 10.20900/jpbs.20210020

**Published:** 2021-10-27

**Authors:** Jing Ma, Jun Ding, Jiawen Hu, Kai Wang, Shuaijun Xiao, Ting Luo, Shuxiang Yu, Chuntao Liu, Yunxuan Xu, Yingxian Liu, Changhong Wang, Suqin Guo, Xiaohua Yang, Haidong Song, Yaoguo Geng, Yu Jin, Huayun Chen, Chunyu Liu

**Affiliations:** 1 Department of Child and Adolescent Psychiatry, Brain Hospital of Hunan Province (School of Clinical Medicine, Hunan University of Chinese Medicine), Changsha 410007, Hunan, China; 2 Department of Social Work, Shenzhen Mental Health Center, Shenzhen 518046, Guangdong, China; 3 Futian hospital for prevention and treatment of chronic disease, Shenzhen 518017, Guangdong, China; 4 Xiangyifurong Middle School of Changsha, Changsha 410011, Hunan, China; 5 The Second Affiliated Hospital of Xinxiang Medical University, Xinxiang 453099, Henan, China; 6 Changsha Changjun Bilingual Experimental Middle School, Changsha 410002, Hunan, China; 7 Mental Health Center, Zhejiang University school of Medicine (Hangzhou Seventh People’s Hospital), Hangzhou 310013, Zhejiang, China; 8 School of Education, Zhengzhou University, Zhengzhou 45001, Henan, China; 9 School of Public Health, Sun Yat-sen University, Guangzhou 510970, Guangdong, China; 10 Division of Epidemiology and Biostatistics, School of Public Health, University of Illinois at Chicago, Chicago, IL 60612, USA; 11 Department of Psychiatry, SUNY Upstate Medical University, New York, NY 13210, USA

**Keywords:** novel coronavirus, psychological state, child and adolescent, anxious symptoms, depressive symptoms, compulsive symptoms

## Abstract

In light of the novel coronavirus’s (COVID-19’s) threat to public health worldwide, we sought to elucidate COVID-19’s impacts on the mental health of children and adolescents in China. Through online self-report questionnaires, we aimed to discover the psychological effects of the pandemic and its associated risk factors for developing mental health symptoms in young people.

We disseminated a mental health survey through online social media, WeChat, and QQ in the five Chinese provinces with the most confirmed cases of COVID-19 during the late stage of the country-wide lockdown. We used a self-made questionnaire that queried children and adolescents aged 6 to 18 on demographic information, psychological status, and other lifestyle and COVID-related variables.

A total of 17,740 children and adolescents with valid survey data participated in the study. 10,022 (56.5%), 11,611 (65.5%), 10,697 (60.3%), 6868 (38.7%), and 6225 (35.1%) participants presented, respectively, more depressive, anxious, compulsive, inattentive, and sleep-related problems compared to before the outbreak of COVID-19. High school students reported a greater change in depression and anxiety than did middle school and primary school students. Despite the fact that very few children (0.1%) or their family members (0.1%) contracted the virus in this study, the psychological impact of the pandemic was clearly profound. Fathers’ anxiety appeared to have the strongest influence on a children’s psychological symptoms, explaining about 33% of variation in the child’s overall symptoms. Other factors only explained less than 2% of the variance in symptoms once parents’ anxiety was accounted for.

The spread of COVID-19 significantly influenced the psychological state of children and adolescents in participants’ view. It is clear that children and adolescents, particularly older adolescents, need mental health support during the pandemic. The risk factors we uncovered suggest that reducing fathers’ anxiety is particularly critical to addressing young people’s mental health disorders in this time.

## BACKGROUND

The novel coronavirus (COVID-19) is highly contagious. On August 23, 2020, there were 84,967 confirmed cases of COVID-19 infections in China and 23,057,288 worldwide [1,2]. Thus far, 4634 people in China and 800,906 in total have died as a result of the virus, and children and adolescents are among the people that it continues to kill [3].

To prevent the spread of the epidemic, 31 provinces and cities in China have implemented strict prevention and control measures, such as blockading cities and shutting down schools. On January 17, 2020, more than 220 million children and teenagers have been confined to their homes after the Ministry of Education of China issued a nationwide notice to postpone the start of the 2020 spring semester for institutions of primary and secondary schools and kindergartens [4,5]. From mid-February 2020, students across the country began online learning. The panic about the epidemic, home confinement, prolonged school closure, and the resulting changes in the way of life and learning may harm the mental health of children and adolescents [5,6]. This may increase the risk of emotional distress, social isolation, disruption of sleep-wake, and interference of physical exercise [7–9]. As a result, children and adolescents show more anxious, depressive symptoms, and sleep problems than that before the pandemic. So, it is meaningful and necessary to pay attention to the mental health of children and adolescents during this period and explore the influencing factors.

Previous studies have found that age [10,11], distance learning [10], screen usage [9,12], parents’ occupation as doctors [13], and parents’ mental health during the epidemic [12], the children’s mental health before the outbreak [13,14], high school students [15,16], sleep time [12,16,17], exercise time [12,17,18] affected children’s and adolescents’ mental health. There are some representative studies. Wen Li et al. [12] conducted an online survey of mental health and risk factors among children ages 3 to 12 in 28 provinces of the Chinese mainland. The Strengths and Difficulties Questionnaire (SDQ) was used to assess the child’s mental health problems. They found that the increased mental health problem was independently related to sleep disturbance, physical activity < 1 h /day, media exposure ≥ 2 h /day, poor parental mental health. A study of the prevalence and risk factors of depression among female adolescents during the COVID-19 outbreak also found a strong correlation between sleep time, physical activity time, and the incidence of depression among adolescents [19]. In the study, depressive symptoms was assessed using the Center for Epidemiologic Studies (CES-D). All of these studies used standardized tools to measure the mental state or depression of children and adolescents during the outbreak of COVID-19. Those already validated tools does not directly reflect the impact of COVID-19 on the child’s mental health, since they investigate the child’s current mental health status, there was no comparison of the psychological state of the children before and after the outbreak of the COVID-19. If the children in the study had been depressed or anxious before the outbreak of the COVID-19, the current psychological state of the children was also depressed or anxious using the standardized scale, in this way it was impossible to determine whether the children’s depressive or anxious state were caused by the occurrence of the epidemic. So we used a self-designed questionnaire to investigate the mental health problems of children and adolescents caused by the epidemic and its influencing factors.

Based on the research and clinical reports of children and adolescents with psychological disturbances that have been done thus far during COVID-19, we hypothesize that: (1) many young people reported more depressive and anxious symptoms, as well as a decrease in concentration and an increase in sleeplessness compared to before the outbreak; (2) during the pandemic, middle school students have reported more of the aforementioned symptoms than primary school students; (3) mandatory quarantine, children’s perception of high parental anxiety about COVID-19, low intensity of exercise, parental and own infection status, and having parents who work in a medical field are positively associated with anxious and depressive symptoms in children. We performed a large online survey to collect data to test these hypotheses.

## METHODS

### Participants

Students aged six to eighteen were the main subjects of the study. They came from the five Chinese provinces that were the hardest hit by COVID-19: Hunan, Hubei, Guangdong, Zhejiang, and Henan.

### Ethics Approval

This study has been approved by ‘Ethics Committee of Shenzhen Futian District Chronic Disease Prevention and Treatment Hospital’ on February 10, 2020. The IRB waived the need for the parenťs consent since it is an online anonymous survey. No privacy-related information, like name, address, etc. will be collected.

### Measures

An online questionnaire (https://www.wjx.cn/) was used to create and publish the self-made questionnaire. Social media, WeChat, and QQ (They are social software, and the most widely used in China) were used to disseminate the questionnaires, which participants completed between February 19th and March 5th. During this period, most parts of China were still in complete lockdown, which started on January 23rd and ended around late April in 2020.

Questions about mental symptoms were written in language that children could understand. When the children had difficulties reading questions or responding, their parents were permitted to help them.

### Questionnaire

The questionnaire contained two parts, one of which included demographic and lifestyle information, and the other of which asked about psychological status of self and parents.

Demographic and lifestyle information included questions about age, place of residence, mental disorder history, whether or not family members had been infected, quarantine status, whether participants had had close contact with an infected person, parents’ profession (medical staff member or not, fever clinic staff member or not), parents’ risk of contracting COVID-19, as well as duration and intensity of participant exercise.

The section on psychological status included questions about whether the participant’s parents were more anxious than before the outbreak of COVID-19 in the child’s opinion, as well as whether the children felt more anxious, depressive, compulsive, irritable, and unable to focus and sleep compared to before the outbreak. Children were asked about three anxiety symptoms: inability or difficulty relaxing muscles; excessive worries; and nervousness, fidgeting, and restlessness. Depressive symptoms included four symptoms: low mood; diminished interest; helplessness; and irritability. Compulsive symptoms include compulsive hand-washing and compulsive thinking. Scoring options for the second part of the survey ranged from zero to ten, with a zero indicating the child’s experience of the symptoms were the same as before the outbreak and a ten indicating their symptoms were the worst they had ever reported.

### Quality Control

The survey used a web-based questionnaire, and only one response could be given per IP address. To be included in the study, participants needed to: (1) be between the ages of six and eighteen; (2) have completed the questionnaire in more than one minute; (3) resided in one of the five following provinces: Hubei, Hunan, Henan, Zhejiang, or Guangzhou. All other participants were excluded. Participants were also excluded if their responses did not match the questions. We initially collected 20,677 questionnaires; after some responses were eliminated according to the aforementioned exclusion criteria, 17,740 valid questionnaires remained.

### Statistical Analyses

Data were analyzed using SPSS version 25.0. Descriptive statistics were obtained first. Logistic regression analyses were then used to compare the risk of anxious symptoms, depressive symptoms, compulsive symptoms, inattentive symptoms, and sleep problems between primary, middle, and high school students, where odds ratios were calculated for high school students and middle school students with respect to primary school students. To examine factors influencing children’s psychological state during the COVID-19 outbreak, a principal component analysis of the outcomes, including anxious, depressive, and compulsive symptoms, was performed. The first principal component captured 65% of variation in outcomes. The first principal component was used as the major dependent variable in the subsequent multivariate linear regression analysis of the risk factors. The independent variables, (e.g., mental disorder, mandatory isolation, exercise intensity), were examined for their effect on children’s psychological state during the COVID-19 outbreak. Supplemental analyses of the residual scores after the first principal component had been removed from the outcomes were also performed using the factor analysis and multivariate regression analyses. In all the analyses, we use a *p*-value of 5% as the cutoff in analyzing significance of risk factors.

## RESULTS

### Characteristics of the Study Population

A total of 17,740 children and adolescents participated in the study. Table 1 lists summary statistics of the study sample.

Tables 2 and 3 display the summary statistics of the impact of COVID-19 on children’s psychological symptoms in their eyes. Table 3 shows that, among the participants, 10,022 (56.5%), 11,611 (65.5%), 10,697 (60.3%), 6868 (38.7%), and 6225 (35.1%), respectively, presented more depressive, anxious, compulsive, inattentive, and sleep problems than before the outbreak of COVID-19.

### The Severity of Psychological Symptoms Increases with Age: Comparison of the Impact on Children from Primary, Middle and High Schools

Table 4 shows the results of the logistic regression analyses measuring the association between psychological symptoms and school level. As depicted in Table 3, there was no difference between primary and middle school students on anxiety and depression, or on problems with sleep and attention (*p* > 0.05), but there were differences between primary and high school students (*p* < 0.05). High school students reported more anxiety, depression, attention deficit, and sleep problems than primary school students (OR high school students > 1, OR primary school students = 1). There were no differences between compulsive symptoms in high school students, middle school students, and primary school students (*p* > 0.05).

### Factors Influencing Children’s Psychological State during the COVID-19 Outbreak

Since depressive symptoms, anxious symptoms, and compulsive symptoms measure some similar psychological features, we first performed a principal component analysis. The results are displayed in Tables 5 and 6.

The first principal component explained 65.45% of the total variation of all the outcomes. In comparison, the second principal component only accounted for 7% of the total variation (Table 5). The first principal component explained an approximately equal proportion of variance for each of the standardized outcome variables (see Table 6). To make the results more interpretable, total standardized symptom score was used instead of the first principal component in the following regression analyses.

Table 7 displays the results of the linear regression, where the total score is the outcome and the covariates were selected using the stepwise variable selection approach. Interestingly, father’s anxiety about the virus contributed substantially to a child’s psychological symptoms (*R*^2^ = 0.33) while, in comparison, mother’s anxiety about the virus only had a small additional effect on the child’s psychological symptoms (*R*^2^ = 0.02) once the father’s anxiety was accounted for. Although the father’s anxiety was correlated with the mother’s anxiety, it appears that the father’s anxiety had a stronger influence on a child’s psychological symptoms than the mother’s anxiety.

Other factors that had significant effects on the child’s psychological symptoms included age, history of mental disorders, mandatory quarantine status, whether their mother worked as a medical staff employee, exercise intensity, and whether a parent had been infected with COVID-19 to the child’s knowledge. However, these factors explained less than 2% of the variation in the child’s symptoms once parents’ anxiety was accounted for. Given the huge sample size in this study, the importance of these significant results that add little interpretive power (*R*^2^) can be limited.

To examine if there were any important risk factors that explained the remaining variance in the child’s symptoms other than the total score, we examined the residual scores obtained by projecting the outcomes to the orthogonal complement space of the total score. Factor analysis was performed on the residual scores, and regression analyses were then performed using the identified factors as outcomes and the risk factors as covariates. The results of these analyses suggest that the covariates do not explain a major proportion of the residuals. The results of these additional analyses are provided in the supplemental materials.

## DISCUSSION

In this study, we explored levels of psychological distress since the COVID-19 pandemic in 17,740 children and adolescents. The six-to-eighteen-year-old students, all of whom resided in the five Chinese provinces with the highest number of reported cases, responded to questionnaires regarding whether their depressive, anxious, compulsive, inattentive, and sleep-related issues had heightened since the beginning of the pandemic.

More than half of the participants reported more anxious and depressive symptoms than they had before the outbreak, and about one-third of the participants reported more sleeplessness and inattentive symptoms. Though reports of compulsion were approximately equal across primary school, middle school, and high school students, we found that high school students reported significantly more worsening of anxiety, depression, inattention, and trouble with sleep than did primary school students. Previous research has found that grade level is positively correlated with depression and anxiety symptoms [20,21]. Additionally, because our study asked participants about their experience of symptoms as compared to their normal state, our results suggests that, when compared to younger children, adolescents’ mood and anxiety are more susceptible to change as a result of the current pandemic conditions (as opposed to simply having a higher baseline rate of depression and anxiety).

Since COVID-19, the rates of anxiety and depression in Chinese adults have been reported at 6.33%–32.1% and 16.5%–17.17%, respectively [21,22], and 57.1% have reported poor sleep [23]. 43.7% of Chinese high school student have been experiencing depressive symptoms, and 37.4% have been experiencing anxiety symptoms [20]. The reported prevalence of depressive symptoms among children and adolescents in China during the pandemic have been somewhat lower than for adolescents, at 22.28% [24]. In contrast, during non-epidemic periods, the prevalence of depression and anxiety in China was 6.8% and 7.6% in the general population [25], and ~3.5%–44.0% for depression and ~20.31%–26.70% for anxiety in children and adolescents [25]. Because only a small proportion of the children in this study had confirmed COVID-19 diagnoses, it appears their psychological symptoms were caused primarily by the psychological distress around the conditions coinciding with the pandemic, not the health effects of the virus itself.

When we examined the relationship between independent variables and degree of psychological distress, we found that the children’s perception of their fathers’ anxiety about COVID-19 explained a sizable proportion (~33%) of variance in reported changes in psychological distress, while perception of mothers’ anxiety they perceived explained only a small proportion of variance after father’s anxiety they perceived was accounted for. This relationship between children’s self-rated total scores of psychological symptoms and the belief that their parents were anxious about the pandemic has not been reported before, nor was this trend reported during SARS. Though we cannot draw a definitive directional or causal conclusion between the two factors or know whether the children’s perceptions were accurate, it is possible that father’s anxiety about COVID-19 in children’s eyes had more of an impact on children’s depressive and anxious symptoms than that of their mothers because mothers are usually more sensitive and their emotions change more frequently than fathers’ emotions [26,27], which could lead children to become desensitized to their mothers’ emotions. In contrast, fathers usually do not visibly display anxiety [26,27], which could make children more likely to sense it in their fathers when it is displayed. The fathers’ stronger impact on children’s emotion was reported before [28,29]. Previous research has addressed the point that when times are tough, parental support can ease children’s stress and anxiety, but amidst the outbreak of COVID-19, parents have also been feeling a lot of anxiety due to the risk of infection and financial stress. This likely leaves parents with fewer emotional resources with which to support their children. As a result, children’s perception of their parents’ anxiety could in turn increase their own anxiety [30].

Age of participant, history of mental disorders, mandatory quarantine status, whether the participant’s mother worked as a medical staff member, exercise intensity, and parent infection status had significant effects on the child’s psychological symptoms but, in total, still explained less than 2% of the variation in symptoms once parents’ anxiety was accounted for. Some of these findings were consistent with previous studies in adults [31–35] and children (specifically with regard to effects of age [24], quarantine status [22,36,37], mother’s profession [24], and having a family member infected with COVID-19 [38]). Some research has found that participants with a history of mental disorders were more vulnerable to stressful events and likely to have more emotional problems than the general population during the pandemic, though these studies used relatively qualitative methods [39,40]; many previous studies have suggested that lower exercise intensity is related to more symptoms of depression and anxiety in adolescents, though these studies were conducted outside the context of the COVID-19 pandemic [41–44].

Though the significant independent variables, aside from paternal anxiety, only explain a small proportion of variance in children’s psychological wellbeing after parental anxiety is accounted for, they reveal interesting connections between mental health of youth and life factors in an unusual historical moment.

The findings above have important clinical implications. For example, we found evidence that children and adolescents, especially those with a history of mental disorders, may need more psychological attention and care during a public health emergency such as the one that the world is currently experiencing. Additionally, management of parents’ (especially fathers’) own anxiety may help their children’s mental wellbeing.

One limitation of the current study is the fact that all participants filled in the questionnaire online without supervision, though rigorous quality control was implemented. Additionally, the sample size collected in Hubei, the hardest-hit area, is the smallest in this study. The respondents in Hubei may not represent subjects in the area well. The gender as a based data havenʼt been collected, moreover, long lasting outcomes have not been considered because it was not a follow-up study. The participants need to look back and answer the questions after comparing status before and after the pandemic. It may cause bias during this process.

In conclusion, the COVID-19 outbreak has significantly influenced Chinese children’s and adolescents’ psychological states in participants’ view. More than half of the participants reported more anxious and depressive symptoms than before, and about one-third of the participants had more sleepless and inattentive symptoms. With respect to their baseline states of mood and anxiety, adolescents were more anxious than younger children, and high school students were more depressive and anxious than middle school and primary school students. Father’s perceived anxiety about the virus had a substantially stronger influence on a child’s psychological symptoms than mother’s anxiety did. Other factors, including age, history of mental disorders, mandatory quarantine status, whether the participant’s mothers worked as a medical staff member, exercise intensity, and whether the child’s parents had been infected with COVID-19, were also found to have significant effects on the child’s psychological symptoms. Still, when added together, they explained less than 2% of variances in the child’s symptoms once parents’ anxiety was accounted for.

The crucial takeaways from our study suggest that, though both have been affected, the COVID-19 pandemic has had a heavier impact on adolescent mental health than it has on mental health of younger individuals in participants’ view and that paternal anxiety about the pandemic is heavily related to children and adolescents’ psychological states. Future research should further investigate other mechanisms that may be moderating parental and children’s anxiety, as well as whether paternal anxiety precedes the child’s anxiety about COVID-19 or vice versa. Then, researchers will better be able to work towards mitigating the effects of mental health issues in high-anxiety families within the context of large crises such as the current pandemic.

## Supplementary Material

Supplemental File 1

Supplemental File 2

Supplemental File 3

## Figures and Tables

**Table 1.** Basic sociodemographic statistics of participants (*N* = 17,740).

Variables	$\bar{x}\pm s/n$ (%)

**Age**	12.870 ± 2.652

**Grade**	

primary school students	8182 (46.1%)
middle school students	6543 (36.9%)
high school students	3015 (17.0%)

**Regional distribution**	

Hubei province	305 (1.7%)
Zhejiang province	1314 (7.4%)
Henan province	3541 (20.0%)
Hunan province	4779 (26.9%)
Guangdong province	7801 (44.0%)

**Infection of COVID-19**	

Infected participants	13 (0.1%)
have an infected family member	12 (0.1%)
have a family member who passed away from COVID-19	1 (0.01%)

**The situation of quarantine**	

voluntary home quarantine	15,444 (87%)
quarantined by order of the government, hospitals, and communities	1201 (6.8%)

**Table 2.** The incidence and mean score for different psychological symptoms (*N* = 17,740).

Symptoms	Symptoms Worsened during COVID?	*n* (%)	Mean (95%CI)

Muscle Tension	Yes	8742 (49.3%)	1.63 (1.59–1.66)
	No	8998 (50.7%)	

Excessive worries	Yes	10014 (56.4%)	1.91 (1.88–1.95)
	No	7726 (43.6%)	

Fidgeting	Yes	9096 (51.1%)	1.74 (1.71–1.78)
	No	8671 (48.9%)	

Low mood	yes	8168 (46%)	1.55 (1.52–1.58)
	no	9572 (54%)	

diminished interest	yes	7066 (39.8%)	1.39 (1.35–1.42)
	no	10674 (60.2%)	

Despair	yes	5589 (31.5%)	1.04 (1.01–1.07)
	no	12151 (68.5%)	

Excessive hand washing	yes	9357 (52.7%)	1.66 (1.62–1.69)
	no	8383 (47.3%)	

Compulsive thoughts	yes	8506 (47.9%)	1.35 (1.32–1.38)
	no	9234 (52.1%)	

Quick to lose temper	yes	6420 (36.2%)	1.15 (1.12–1.18)
	no	11320 (63.8%)	

Inattention	yes	6868 (38.7%)	1.25 (1.22–1.28)
	no	10872 (61.3%)	

Sleep problems	yes	6225 (35.1%)	1.29 (1.26–1.32)
	no	11515 (64.9%)	

**Table 3.** The incidence of psychological symptoms among high school, middle school, and primary school students (before versus after the outbreak) (*N* = 17,740).

More mental Symptoms than before	Primary school students(*n* = 8182)	Middle school students(*n* = 6543)	High school students(*n* = 3015)

Depressive symptoms	4552 (55.6%)	3670 (56.0%)	1800 (59.7%)
Anxious symptoms	5252 (64.2%)	4284 (65.5%)	2075 (68.8%)
Compulsive symptoms	4933 (60.%3)	3950 (60.4%)	1814 (60.2%)
Inattention	3113 (38.0%)	2452 (37.5%)	1303 (43.2%)
Sleep problems	2871 (35.0%)	2207 (33.7%)	1147 (38.0%)

**Table 4.** Comparison between school grade levels and psychological symptoms.

Variables		Primary school students	Middle school students	High school students

Depressive symptoms	OR	1	1.019	1.181
	95% CI	-	0.954–1.088	1.085–1.286
	P	-	0.58	<0.001

Anxious symptoms	OR	1	1.058	1.231
	95% CI	-	0.988–1.133	1.126–1.347
	P	-	0.105	<0.001

Compulsive symptoms	OR	1	1.003	0.995
	95% CI	-	0.939–1.072	0.913–1.083
	P	-	0.922	0.905

Inattention	OR	1	0.976	1.239
	95% CI	-	0.913–1.044	1.139–1.349
	P	-	0.477	<0.001

Sleep problems	OR	1	0.942	1.136
	95% CI	-	0.879–1.008	1.042–1.238
	P	-	0.085	<0.001

**Table 5.** Characteristic values of input data, cumulative variance contribution rate and KMO and Bartlett’s Test.

Component	Total	Variance (%)		Cumulative Variance (%)

1	7.200	65.451	65.451	
2	0.774	7.040	72.491	
3	0.642	5.837	78.328	
4	0.459	4.170	82.498	
5	0.407	3.702	86.201	
6	0.336	3.056	89.257	
7	0.312	2.832	92.089	
8	0.258	2.342	94.431	
9	0.234	2.130	96.561	
10	0.201	1.828	98.389	
11	0.177	1.611	100.000	

Kaiser-Meyer-Olkin Measure of Sampling Adequacy				0.946
Bartlett’s Test of Sphericity				<0.001

**Table 6.** Principal component analysis results.

Variable	Variance Explain by Component 1

Low mood	0.862
Excessive worries	0.858
Fidgeting	0.824
diminished interest	0.823
Despair	0.822
Compulsive thoughts	0.820
Inattention	0.814
Easy to lose temper	0.803
Muscle Tension	0.800
Excessive hand washing	0.736
Sleep problems	0.726

**Table 7.** Analysis of factors influencing total score.

Step	Variable	*R* ^2^	Adjusted *R*^2^	*R*^2^ change	Change in *F*-value	Coefficient	*P*-value	Collinearity Statistics	
								Tolerance	VIF

-	(Constant)	-	-	-	-	−0.667	<0.001	-	
1	Father’s anxiety about the virus	0.331	0.331	0.331	8776.52	0.128	<0.001	0.247	4.056
2	Mother’s anxiety about the virus	0.347	0.347	0.016	434.239	0.086	<0.001	0.247	4.053
3	Mental disorder history	0.349	0.349	0.002	52.244	0.67	<0.001	0.987	1.013
4	Age	0.350	0.350	0.001	23.131	0.01	<0.001	0.995	1.005
5	Forced quarantine	0.350	0.350	0.001	17.849	0.098	<0.001	0.994	1.006
6	Mothers’ profession^a^	0.351	0.351	0.000	12.631	−0.129	<0.001	0.998	1.002
7	Exercise intensity	0.351	0.351	0.000	7.745	−0.031	0.005	0.998	1.002
8	PCD^b^	0.351	0.351	0.000	7.429	0.638	0.006	0.987	1.013

Durbin-Watson						1.08			
Dependent variable: Total standardized symptom score									

a: Whether the participant’s mother was in a medical profession or not.

b: Parental COVID-19 diagnosis.
