# Supplementary material for: Children and Adolescents’ Psychological Well-Being Became Worse in Heavily Hit Chinese Provinces during the COVID-19 Epidemic
Source: J Psychiatr Brain Sci. Author manuscript; Available in PMC 2021 Dec 8. (PMC8653505; doi:10.20900/jpbs.20210020)
Supplement: Supplemental File 1 [file NIHMS1752736-supplement-Supplemental_File_1.pdf]

医学伦理审查报告

我院拟开展“新型冠状病毒肺炎（COVID-19）期间儿童青少年心理健康状况及影响因素的横断面调查”的科研工作，我院伦理委员会对该项目相关医学伦理学问题进行了审查。

项目信息：

项目类型：项目

项目名称：新型冠状病毒肺炎（COVID-19）期间儿童青少年心理健康状况及影响因素的横断面调查

承担单位：深圳市福田区慢性病防治院

项目负责人： 王凯

研究起止时间：2020.2.19-2020.03.07

涉及人（或动物实验）的相关研究主要内容介绍：

此次研究使用的是自制的网络调查问卷，通过问卷星(www.wjx. cn)来创建和发布，通过微信和QQ等社交软件来传播。2月19日到3月7日之间，所有问卷将由参与者本人进行填写。

调查问卷包括人口学信息和心理状况两部分。

人口统计资料包括：年龄、居住地点、精神病史、家庭成员是否感染、是否被隔离或曾与感染者有密切接触、父母的职业(是否医务人员、是否有发烧门诊、感染2019冠状病毒疾病的风险)、运动时间和强度。

心理状态包括：父母是否比冠状病毒疾病爆发前更焦虑；孩子是否比疫情暴发前感到更多的焦虑、抑郁、强迫、易怒等症状以及无法集中注意力与眠差的问题。

此项研究使用的是无接触的网上在线调查，所收集的信息与数据只用于本

实验研究，且严格遵循保密原则。

审查评议意见：

经我院伦理委员会审议，该研究的实验设计和实施方案充分考虑了安全性和公平性原则，研究内容不构成对受试者的伤害和风险，受试者的招募将基于自愿和知情同意原则，并尽最大限度保护受试者的隐私。

结论：在该研究中，受试者权益得到充分保护，对受试者不存在潜在风险，同意该项目的现场工作按计划进行。

深圳市福田区慢性病防治院伦理委员会

202[illegible]10 日

深圳市福田区慢性病防治院 伦理委员会

伦理委员会专家签名：

[illegible] [illegible] [illegible] [illegible]

[illegible] [illegible] [illegible] [illegible]

Psychological status includes whether the parents were more anxious than before the outbreak of COVID-19 in children's eyes. Whether the children felt more anxious, depressive, compulsive, irritable,unable to focus, and sleep problems than before the outbreak.

The information and data collected in the study, which used a contactless online survey, were used only for the study and were strictly confidential.

Review of comments:

After deliberation by the Ethics Committee of our hospital, we think that the design and implementation scheme of the study took full account of the principles of safety and fairness, and the contents of the study will not harm the subjects. Participants will be recruited on the basis of the principle of voluntary and with the utmost protection of their privacy.

Conclusion: The rights and interests of the subjects are fully protected in this study. There is no potential risk to the subjects, and this project is agreed to proceed as planned.

Organization： Ethics Committee of Shenzhen Futian District Chronic Disease Prevention and Treatment Hospital

Time： February 10,2020
