# Supplementary material for: Children and Adolescents’ Psychological Well-Being Became Worse in Heavily Hit Chinese Provinces during the COVID-19 Epidemic
Source: J Psychiatr Brain Sci. Author manuscript; Available in PMC 2021 Dec 8. (PMC8653505; doi:10.20900/jpbs.20210020)
Supplement: Supplemental File 2 [file NIHMS1752736-supplement-Supplemental_File_2.pdf]

SUPPLEMENTARY MATERIAL

The residuals for the total symptom scores (i.e., the projection of the outcomes to the orthogonal complement space of the total score) were examined. A factor analysis was first performed on the residual scores and regression analyses were then performed using the identified factors as outcomes and the risk factors as covariates.

The average score, C1= (ZX1+ZX2 +ZX3+ZX4+ZX5+ZX6+ZX7+ZX8+ZX9 +ZX10+ZX11)/11, was used in the first regression analysis. Next, we used ZX1 as the outcome and C1 as the covariate, ran a univariate regression, and obtained the residuals of the regression, denoted WX1. We repeated the same regression analysis with ZX1 replaced by ZX2, ZX3, etc. in subsequent regressions to obtain the corresponding residuals, denoted WX2, WX3, etc. We then used the residuals (i.e., WX1, WX2 ..., WX11) as the inputs for the factor analysis. In short, we kept the first interpretable component (slightly distinct from the first principal component) fixed and rotated the rest of the components.

The results of the factor analysis and regression analysis of residuals WX1-WX11 are as follows:

Total Variance Explained

Component	Initial Eigenvalues			Rotation Sums of Squared Loadings		
	Total	% of Variance	Cumulative %	Total	% of Variance	Cumulative %
1	2.213	20.115	20.115	1.959	17.811	17.811
2	1.818	16.530	36.645	1.670	15.184	32.995
3	1.278	11.621	48.266	1.443	13.120	46.115
4	1.074	9.764	58.030	1.311	11.915	58.030
5	1.013	9.213	67.243			
6	.956	8.690	75.933			
7	.720	6.548	82.481			
8	.686	6.232	88.713			
9	.678	6.162	94.876			
10	.564	5.124	100.000			
11	9.283E-15	8.439E-14	100.000			

Rotated Component Matrix[a]

	Component			
	1	2	3	4
WX Loses temper easily	-.741	.140	0.012	-.233
WX Muscle tension	.715	.121	-.037	-.321
WX Worries too much	.693	.113	.131	-.392
WX Inattention	-.616	.175	-.306	-.250
WX Excessive hand washing	.065	-.779	-.054	-.008
WX Excessive thinking	-.009	-.774	-.063	-.057
WX Sleep problems	-.140	.270	-.693	.051
WX Low spirits	-.031	.308	.667	.230
WX Fidgeting	.100	.355	.630	-.267

WX Loss of interest	.042	.289	-.029	.651
WX Despair	-.058	-.094	.013	.620

Extraction Method: Principal Component Analysis.

Rotation Method: Varimax with Kaiser Normalization.

a. Rotation converged in 7 iterations.

Table S1 Regression analysis of the first principal component on the covariates

step	Variable	R2	Adjusted R2	R2 change	F change	B	P	Tolerance	VIF
0	（Constant）					-0.341	<0.001		
1	Father's nervousness	0.051	0.051	0.051	957.08	0.052	<0.001	0.246	4.06
2	Mother's nervousness	0.053	0.053	0.002	36.922	0.032	<0.001	0.246	4.059
3	Exercise duration	0.055	0.055	0.002	32.907	0.033	<0.001	0.893	1.12
4	PD[a]	0.056	0.056	0.001	19.464	-0.516	<0.001	0.999	1.001
5	PR[b]	0.056	0.056	0	7.513	-0.071	0.007	0.994	1.006
6	Neighbor status[c]	0.057	0.056	0	4.227	-0.076	0.034	0.995	1.005
7	Exercise intensity	0.057	0.056	0	3.859	0.028	0.049	0.894	1.119
Durbin-Watson					1.71				
Dependent variable: First Principal Component									

a: history of physical disorder

b: risk of infection in parents

c: neighbor's COVID-19 infection status

Table S2 Regression analysis of the second principal component on the covariates

step	Variable	R2	Adjusted R2	R2 change	F change	B	P	Tolerance	VIF
0	(Constant)					0.06	0.148		
1	Exercise time	0.006	0.006	0.006	112.454	-0.058	<0.001	0.887	1.128
2	Age	0.009	0.008	0.002	40.004	0.016	<0.001	0.988	1.012
3	Father nervous	0.01	0.01	0.001	20.366	-0.038	<0.001	0.246	4.058
4	Mother's nervousness	0.011	0.011	0.002	28.713	0.028	<0.001	0.247	4.054
5	Exercise intensity	0.012	0.012	0.001	18.221	-0.062	<0.001	0.895	1.117
6	PD[a]	0.013	0.013	0.001	9.987	0.387	0.002	0.999	1.001
Durbin-Watson					1.96				
Dependent variable: The second Principal Component									

a: history of physical disorder

Table S3 Regression analysis of the third principal component on the covariates

step	Variable	R2	Adjusted R2	R2 change	F change	B	P	Tolerance	VIF
0	(Constant)					-0.021	0.051		
1	Mother's nervousness	0	0	0	7.36	0.007	0.007	1	1
Durbin-Watson					1.992				
Dependent variable: The Third Principal Component									

Table S4 Regression analysis of the fourth principal component on the covariates

step	Variable	R2	Adjusted R2	R2 change	F change	B	P	Tolerance	VIF
0	(Constant)					0.308	<0.001		
1	Mother's nervousness	0.013	0.013	0.013	227.33	-0.039	<0.001	0.998	1.002
2	Age	0.014	0.014	0.001	19.493	-0.012	<0.001	0.999	1.001
3	PCD[a]	0.014	0.014	0	6.485	0.735	0.01	1	1
4	Exercise intensity	0.014	0.014	0	6.109	-0.034	0.013	0.999	1.001
Durbin-Watson					1.978				
Dependent variable: The Fourth Principal Component									

a: Parent COVID-19 diagnosis status

The results of these analyses suggest that the covariates do not explain a major proportion of the residuals.
