# Supplementary material for: Children and Adolescents’ Psychological Well-Being Became Worse in Heavily Hit Chinese Provinces during the COVID-19 Epidemic
Source: J Psychiatr Brain Sci. Author manuscript; Available in PMC 2021 Dec 8. (PMC8653505; doi:10.20900/jpbs.20210020)
Supplement: Supplemental File 3 [file NIHMS1752736-supplement-Supplemental_File_3.pdf]

SUPPLEMENTARY MATERIAL

新冠病毒肺炎疫情对中小学生心理健康状况的影响

The influence of the outbreak of the COVID-19 on the mental health of primary and middle school students.

感谢你阅读本问卷。该问卷将帮助老师、医务工作者、心理工作者及研究者了解突如其来的新冠病毒肺炎疫情对小朋友和青少年心理健康状况的影响，以便社会各界更好地帮助疫情期心理上有困扰的儿童青少年，也可以帮助未来的研究者在类似疫情发生时提前做好准备帮助你的同龄人。

Thanks for reading this questionaire. this questionaire will help teachers, health care workers, psychologists and researchers understand the impact of a sudden outbreak of the COVID-19 on the mental health of children and adolescents, so that people from all walks of life can better help children and adolescents who are psychologically disturbed during the outbreak of the COVID-19. And it can also help researchers prepare for similar outbreaks to help your peers.

如果你是 6 岁至 18 岁的儿童青少年，请继续填写。如果你阅读及或书写有困难，请让爸爸妈妈帮助你阅读，你来回答，再让爸爸妈妈帮你选择和你感受相符的答案。谢谢你哦！

If you are beteween the ages of 6 and 18, please continue. If you have difficulties in reading or writing, please ask your parents help you, they read, and you answer. And then let your parents help you choose the answer that matches your feelings. Thank you!

一、基本信息

Basic informations

1、请问你的年龄是_____岁

How old are you?_____

2、请问你居住在哪个省？ _______

Which province do you live in?

3、 请问你居住在哪个市或县? _______

Which city or county do you live in?

4、在疫情发生之前，你有无被诊断为某种精神障碍(有时候被称作“心理障碍”或者“心理疾病”)？

Have you been diagnosed with a dysphrenia (sometimes be called as "mental disorder" or "metal disease") before the outbreak？

是 YES

否 NO

如果是，请填写：_____

If the answer is yes, please fill in the diagnosis here____

5、你是否被感染新冠肺炎？

Have you contracted the COVID-19?

是 YES

否 NO

6、你身边的家人有被感染新冠肺炎吗？

Is anyone in your family infected?

是 YES

否 NO

7、你的家人有因为感染新冠肺炎去世的吗？
Did anyone in your family died because of the COVID-19?
有 YES
无 NO
8、新冠肺炎疫情发生期间，你和你的家人有被隔离吗？
Were you or your family quarantined durining the outbreak?
是 YES
否 NO
9、你所在的小区有无被确诊的感染者？
Are there any confirmed infections in your community?
是 YES
否 NO
10、你或家人接触过疑似或确诊病例吗？
Have you or your family been exposed to anyone who are suspected or confirmed of being infected?
是 YES
否 NO
11、在你看来你父亲或母亲的工作会有很多机会暴露于病毒感染的风险中吗？（比如医护人员）
In your opinion, do your parents' jobs have many risks to be exposed to viral infections? (such as doctors or nurses)
是 YES
否 NO
12、你父亲的工作是______(如果父亲去世则填“去世”，如果父亲不在身边，请填“不清楚”)
What does your father do?____ (If your father died, please fill in "died". If your father is not with you, please fill in "don't know")
13、你母亲的工作是 _______(如果母亲去世则填“去世”，如果母亲不在身边，请填“不清楚”)
What does your mother do?____(If your mather died, please fill in "died". If your mather is not with you, please fill in "don't know")
14、如果你的父亲/母亲/祖父母是医务人员，他们在疫情期内有无去发热门诊或新冠肺炎隔离病房工作？
If your parents or grandparents are medical staffs, did they worked in the fever clinic or the COVID-19 isolation ward during the outbreak.
有 YES
无 NO
父母均非医务人员 They are not medical staffs.
是医务人员，但是我不清楚有无去这些地方 They are medical staffs but I don't know wheter they went there.

二、疫情期运动、时间使用、疫情相关信息获取与可能出现的精神症状

Exercise, time using, the way to get information about the outbreak and possible psychiatric symptoms during the outbreak.

1、疫情持续期内，你平均每天运动（包括在屋里跳绳、楼下散步等）的时间总计是：
during the outbreak, how long do you exercise on average every day (include rope skipping in the house and walk dowstairs)?
少于半小时
less than half an hour

半至 1 小时
from half an hour to an hour
1 小时至 1.5 小时
1h to 1.5h
1.5 小时至 2 小时
1.5h to 2h
2 小时以上
more than 2h
2、你的运动强度是：
your exercise intensity is:
轻微（如散步）
slight(such like taking a walk)
中等（如快走、跳舞、做家务、打羽毛球）
middle(such like brisk walking, dancing, doing housework, palying badminton)
剧烈（如跑步、快速骑单车、快速游泳）
strenuous(such like running, fast cycling, fast swiming)
3、疫情持续期内，你每天的时间大约是怎么使用的？（可以按照平均数填写）：
during the outbreak, how do you spend your time each day (in average)?
睡觉____小时
sleep for___hour
课内学习___小时
in-class learning for___hour
课外阅读____小时
after-class reading___hour
与家人聊天 ___小时
chat with your family for___hour
运动____小时
exercise for___hour
听音乐 _____小时
listen to music for___hour
发呆、无所事事 _____小时
be in a daze for___hour
刷微信朋友圈 ___小时
browse WeChat moments for___hour
看电视（电影、电视剧、动画片等）___小时
watch TV(movie, TV play, cartoon) for___hour
浏览网页（通过手机或电脑）___小时
browse webpage (via phone or computer) for___hour
玩游戏（通过手机、电脑或电视）____小时
play games (via phone, computer or TV) for____hour
与朋友互动（打电话、聊微信或 QQ）____小时
interact with friends (talk on the phone, WeChat, or QQ) for___hour
提示:总和不大于 24 小时
TIP: the totl is no more than 24 hours

4、疫情持续期内，你每天花费在网络上（包含朋友圈、微信、微博、群、网页、搜索引擎等）搜索、阅读疫情相关的信息的时间大约多久？
during the outbreak, how long do you spend on the internet (include WeChat moments, WeChat, Weibo, group, webpage, search engine)

半小时～1 小时
half an hour to 1h
1 小时～2 小时
1h-2h
2 小时～3 小时
2h-3h
4 小时～5 小时
4h-5h
6 小时或更多
6h and more

5、你每天接受到的全部疫情相关信息大约有多少条？（假设一个朋友圈的微信链接算一条，请选择接近的数量范围）
how many pieces of information do you get every day about the outbreak? (suppose one link from WeChat moments is one information, please chose the approximate quantity)

10 条以下
less than 10
10～20 条
10-20
20～30 条
20-30
30～50 条
30—50
50～100 条
50—100
100 条以上
over 100

6、你每天接受到的疫情相关信息中正面信息大约有多少条？（假设一个朋友圈的微信链接算一条，请选择接近的数量范围）
How many possitive information are there among the information about the outbreak that you received? (suppose one link from WeChat moments is one information, please chose the approximate quantity)

5 条以下
less than 5
5～10 条
5-10
10～20 条
10-20
20～30 条
20-30
30～50 条
30-50

50 条以上
over 50
7、你每天看到的疫情相关信息中负面信息大约有多少条？（假设一个朋友圈的微信链接算一条，请选择接近的数量范围）
How many negative information are there among the information about the outbreak that you received? (suppose one link from WeChat moments is one information, please chose the approximate quantity)
5 条以下
less than 5
5～10 条
5-10
10～20 条
10-20
20～30 条
20-30
30～50 条
30-50
50 条以上
over 50
8、你每天看到的疫情相关信息中中性信息大约有多少条？（假设一个朋友圈的微信链接算一条，请选择接近的数量范围）
How many neutral information are there among the information about the outbreak that you received? (suppose one link from WeChat moments is one information, please chose the approximate quantity)
5 条以下
less than 5
5～10 条
5-10
10～20 条
10-20
20～30 条
20-30
30～50 条
30-50
50 条以上
over 50
9、你每天看到的疫情相关信息中官方信息大约有多少条？（假设一个朋友圈的微信链接算一条，请选择接近的数量范围）
How many official information are there among the information about the outbreak that you received? (suppose one link from WeChat moments is one information, please chose the approximate quantity)
5 条以下
less than 5
5～10 条
5-10
10～20 条
10-20

20～30 条
20-30
30～50 条
30-50
50 条以上
over 50

10、你每天看到的疫情相关信息中非官方信息大约有多少条？（假设一个朋友圈的微信链接算一条，请选择接近的数量范围）
How many unofficial information are there among the information about the outbreak that you received? (suppose one link from WeChat moments is one information, please chose the approximate quantity)

5 条以下
less than 5
5～10 条
5-10
10～20 条
10-20
20～30 条
20-30
30～50 条
30-50
50 条以上
over 50

11、你能分辨你阅读的疫情相关信息的真伪吗？
can you tell the truth from the information you read about the outbreak?

很容易分辨
very easy to tell
容易分辨
easy to tell
有些容易有些困难
a little hard to tell
难以分辨
hard to tell
非常难分辨
very hard to tell

12、在你看来，疫情让你爸爸感到紧张、害怕吗？（如果爸爸不在身边，请选择不适用）（0 分表示没有紧张害怕，10 分表示紧张害怕到极点）
In your opinion, dose the outbreak make your father nervous? (If your father is not with you, please chose Not Applicable)
(0 means not nervous at all, 10 means very nervous)

0 1 2 3 4 5 6 7 8 9 10 不适用
0 1 2 3 4 5 6 7 8 9 10 Not Applicable

13、在你看来，疫情让你妈妈感到紧张、害怕吗？（如果爸爸不在身边，请选择不适用）（0 分表示没有紧张害怕，10 分表示紧张害怕到极点）

In your opinion, dose the outbreak make your father nervous? (If your mather is not with you, please chose Not Applicable)

(0 means not nervous at all, 10 means very nervous)

0 1 2 3 4 5 6 7 8 9 10 不适用

0 1 2 3 4 5 6 7 8 9 10 Not Applicable

14、和疫情发生前相比，你会觉得更加肌肉紧张、难以放松吗？

Compared to before the outbreak, do you feel more muscular tension and difficulty relaxing?

0 分是和疫情前一样，10 分是糟糕非常多，请选择具体分值：

0 means the same as before the outbreak, 10 means much worse.

0 1 2 3 4 5 6 7 8 9 10

15、和疫情发生前相比，你更加会为很多事情过分紧张、担心吗？

Compared to before the outbreak, do you feel more nervous and worried about many things?

0 分是和疫情前一样，10 分是糟糕非常多，请选择具体分值：

0 means the same as before the outbreak, 10 means much worse.

0 1 2 3 4 5 6 7 8 9 10

16、和疫情发生前相比，你有没有总觉得烦躁不安？

Compared to before the outbreak, do you feel more dysphoric?

0 分是和疫情前一样，10 分是糟糕非常多，请选择具体分值：

0 means the same as before the outbreak, 10 means much worse.

0 1 2 3 4 5 6 7 8 9 10

17、和疫情发生前相比，你的情绪更低落吗？

Compared to before the outbreak, do you feel more down?

0 分是和疫情前一样，10 分是糟糕非常多，请选择具体分值：

0 means the same as before the outbreak, 10 means much worse.

0 1 2 3 4 5 6 7 8 9 10

18、和疫情发生前相比，你对以往感兴趣的事情更加没有兴趣吗？

Compared to before the outbreak, do you feel less interested in the things you were interested in?

0 分是和疫情前一样，10 分是糟糕非常多，请选择具体分值：

0 means the same as before the outbreak, 10 means much worse.

0 1 2 3 4 5 6 7 8 9 10

19、疫情有没有让你感到无助绝望？

Dose the outbreak make you feel helpless?

0 分是和疫情前一样，10 分是糟糕非常多，请选择具体分值：

0 means the same as before the outbreak, 10 means much worse.

0 1 2 3 4 5 6 7 8 9 10

20、和疫情发生前相比，你总会担心手没洗干净而感染病毒，从而花费每天超过 1 小时用于洗手吗？

Compared to before the outbreak, do you spend more than an hour a day washing your hands because you're always worried about getting a virus because your hands aren't clean?

0 分是和疫情前一样，10 分是糟糕非常多，请选择具体分值：

0 means the same as before the outbreak, 10 means much worse.

0 1 2 3 4 5 6 7 8 9 10

21、和疫情发生前相比，你每天会花费大量时间？（比如 1 小时以上）思考疫情相关的问题，自己觉得没有必要但是控制不住吗？

Compared to before the outbreak, do you spend a lot of time (like over an hour) each day thingkinng about the outbreak, feeling unnecessary but unable to control it?

0 分是和疫情前一样，10 分是糟糕非常多，请选择具体分值：

0 means the same as before the outbreak, 10 means much worse.

0 1 2 3 4 5 6 7 8 9 10

22、和疫情发生前相比，你有没有更加容易发脾气？

Compared to before the outbreak, do you lose your temper more easily?

0 分是和疫情前一样，10 分是糟糕非常多，请选择具体分值：

0 means the same as before the outbreak, 10 means much worse.

0 1 2 3 4 5 6 7 8 9 10

23、和疫情发生前相比，你有没有更加难以集中注意力？

Compared to before the outbreak, do you feel more difficult to concentrate?

0 分是和疫情前一样，10 分是糟糕非常多，请选择具体分值：

0 means the same as before the outbreak, 10 means much worse.

0 1 2 3 4 5 6 7 8 9 10

24、和平日相比，疫情期你的睡眠怎么样？

Compared to before the outbreak, how's your sleep during the outbreak?

0 分是和疫情前一样，10 分是糟糕非常多，请选择具体分值：

0 means the same as before the outbreak, 10 means much worse.

0 1 2 3 4 5 6 7 8 9 10

三、疫情的其它影响

Other effects of the outbreak

1、、疫情导致无法按时开学，这对你的影响是怎样的？

How dose the outbreak affect you because you can't start school on time?

有积极的影响，在家学习效率比在学校时更高

It has a postive effect. Studying at home is more efficient than at school.

没有影响，在家学习效率和在学校时一样

It has no effect. Studying at home is just as effective as studying in school.

让我有一些担心、焦虑，可以基本正常在家里学习

It makes me feel a little anxious, but I can almost normally study at home.

让我非常焦虑、担心、烦躁，但是我还是能勉强在家学习

It makes feel very anxious, worried, whiny, but I can barely study at home.

我非常焦虑、担心、烦躁，以至于我几乎没有办法在家里学习

It makes feel so anxious, worried, whiny that I can hardly study at home.

2、疫情期在家上网课，你的效率整体怎样？

How's your efficiency in studying on the internet during the outbreak?

非常好

very good

良好

good

一般

so-so

有点差

a little inefficient
非常差
inefficient

3、新冠疫情有没有让你对医学科学或者病毒相关研究产生浓厚的兴趣？

Dose the COVID-19 makes you interested in medical science or virus-related research?
非常多
A lot.
有点多
A little.
一般
So-so.
几乎没有
Barely.
完全没有
Not at all.

4、疫情期看到医务工作者被感染甚至有些死亡，会不会影响你对医护职业的看法？

There were medical workers infected or even died. Will this fact affect your perception of the medical profession?

有影响，未来坚决不当医生护士
YES, I will never be a doctor or nurse in the future.
有影响，我觉得医护很伟大，我想未来从事医护职业
YES, I think the medical works are great, I want to do the medical profession in the future.
没有影响，对于医护职业我的想法和疫情发生前一样，没有改变
NO, my perception of the medical profession is as same as before, not changed.
我不确定有无影响
I'm not sure about that.
